# In Vitro Anti-Inflammatory Effect of *Salvia sagittata* Ethanolic Extract on Primary Cultures of Porcine Aortic Endothelial Cells

**DOI:** 10.1155/2019/6829173

**Published:** 2019-05-09

**Authors:** Irvin Tubon, Augusta Zannoni, Chiara Bernardini, Roberta Salaroli, Martina Bertocchi, Roberto Mandrioli, Diego Vinueza, Fabiana Antognoni, Monica Forni

**Affiliations:** ^1^Department of Veterinary Medical Sciences-DIMEVET, University of Bologna, Ozzano dell'Emilia Bologna 40064, Italy; ^2^Escuela de Bioquimica y Farmacia, Facultad de Ciencias, Escuela Superior Politecnica de Chimborazo, Riobamba, EC060155, Ecuador; ^3^Escuela de Enfermeria, Facultad de Ciencias Medicas, Universidad Regional Autónoma de Los Andes UNIANDES, Ambato, EC180150, Ecuador; ^4^Department for Life Quality Studies-QuVi, University of Bologna, Rimini 47921, Italy

## Abstract

The aim of the present research was to study the effects of an ethanolic extract of *Salvia sagittata* Ruiz & Pav (SSEE), an endemic Ecuadorian plant traditionally used to treat inflammation and different intestinal affections, on primary cultures of porcine aortic endothelial cells (pAECs). pAECs were cultured in the presence of different concentrations (1-200 *μ*g/mL) of SSEE for 24 h, and cytotoxicity was evaluated by the MTT assay. SSEE did not negatively affect cellular viability at any concentration tested. Cell cycle was analyzed and no significant change was observed. Then, the anti-inflammatory effects of SSEE on pAECs were analyzed using a lipopolysaccharide (LPS) as the inflammatory stimulus. Different markers involved in the inflammatory process, such as cytokines and protective molecules, were evaluated by real-time quantitative PCR and Western blot. SSEE showed the ability to restore pAEC physiological conditions reducing interleukin-6 and increasing Heme Oxygenase-1 protein levels. The phytochemical composition of SSEE was also evaluated via HPLC-DAD and spectrophotometric assays. The presence of different phenolic acids and flavonoids was revealed, with rosmarinic acid as the most abundant component. SSEE possesses an interesting antioxidant activity, as assessed through both the Oxygen Radical Absorbance Capacity (ORAC) and 2,2-diphenyl-1-picrylhydrazyl (DPPH) assays. In conclusion, results suggest that SSEE is endowed with an *in vitro* anti-inflammatory effect. This represents the initial step in finding a possible scientific support for the traditional therapeutic use of this plant.

## 1. Introduction

In the last few years, researches aimed to scientifically define the effects of natural products have been growing, not only due to the increasing popularity of plant-based Traditional Medicine but also because it meets the primary health-care needs for the majority of the population in developing countries [1]. Moreover, a huge number of medicinal plants, still not investigated, are available worldwide. Currently, more than 20,000 plant species are used to treat several diseases and are considered as potential reservoirs for new drugs [2]. Recent studies suggest that the historical ethnopharmacological uses of plant-based medicines can represent a useful preliminary screening tool in the field of drug discovery [3].

Ecuador is considered one of the countries with the largest biodiversity in the world. The flora of mainland Ecuador is extremely rich: an estimated total of 17,000 species have so far been recorded [4, 5] and more than 3,000 medicinal plants are used in different native communities living on the highlands of the Ecuadorian Andes [6]. However, in most cases, the preparation, doses, and routes of administration of these herbal remedies are only transferred orally from generation to generation, while scientific information regarding their phytochemical or biological activity is insufficient or lacking [7].

*Salvia* L. (sage) is widely known as the largest genus in the Lamiaceae family, and to date, approximately 980 species have been recognized, most of which are restricted to the New World [8]. Some species of this genus have been used since ancient times as medicinal plants all around the world [9–11]. In addition, chemical constituents of various sage plants were described and comprise different terpenoids, several phenolic compounds, such as simple phenolics and caffeic acid derivatives, flavonoids as well as phenolic diterpenoids [12].

*Salvia sagittata* Ruiz & Pav is an herbaceous perennial plant distributed in Ecuador and Peru. It has yellow-green arrow-shaped leaves and very sticky inflorescences in the apical part of the plant, formed by brilliant blue flowers with a prominent lower lip. Its leaves are commonly prepared either in an infusion to counteract different affections such as spasms, diarrhea, flatulence, fever, influenza, gastritis, stomach pain, cuts, and bumps or by heating with brandy and applying topically to treat rheumatism and articular pain [13–15]. Despite the ethnobotanical information in favor of multiple beneficial health effects of *S. sagittata*, scientific evidence from *in vivo* or *in vitro* studies is still lacking.

In order to test the possible biological activity of *S. sagittata* ethanolic extracts (SSEE), we used endothelial cells as a model system, given their fundamental role in different physiological processes. These cells are normally in dynamic equilibrium with their environment, preventing thrombus formation by the expression and secretion of anticoagulant, antiadhesive, and anti-inflammatory molecules. Nevertheless, in pathologic processes, such as inflammation, infection, or genetic alterations, endothelial cells change their phenotype from a resting to an active function that modulates the complement and coagulation cascades, thrombus formation, inflammation, and innate and adaptive immunity [16, 17]. Endothelial cells are also recognized as key regulators of the inflammatory response controlling adhesion and migration of inflammatory cells as well as resolution of inflammation [18].

Tests were carried out on a primary culture instead of a cell line. Despite their viability and unlimited expansion, cell lines do not preserve various important markers and functions shown *in vivo* [19, 20]. On the contrary, primary cells preserve most of these functions. Due to the biological similarities between swine and human at the anatomic [21], proteomic [22], and genomic level [23], primary cultures of porcine aortic endothelial cells have been used as a suitable *in vitro* model of human ones [24–26], as well as to test the anti-inflammatory activity of phytoextracts [27, 28].

In this context, we decided to evaluate SSEE for its phytochemical, antioxidant, and anti-inflammatory characteristics as related to biological activities in primary cultures of porcine aortic endothelial cells stimulated with a bacterial lipopolysaccharide.

## 2. Materials and Methods

### 2.1. Chemicals and Reagents

Human Endothelial Serum-Free Medium (hESFM), heat-inactivated fetal bovine serum (FBS), antibiotic-antimycotic, Dulbecco's phosphate-buffered saline (DPBS) and phosphate-buffered saline (PBS) were purchased from Gibco-Life Technologies (Carlsbad CA, USA), as previously described [27]. Propidium iodide (PI) was purchased from Miltenyi Biotec (Bergisch Gladbach, Germany). RNase A/T1 and the TRIzol reagent were purchased from Thermo Fisher Scientific (Waltham, MA, USA). RNA isolation was performed with a NucleoSpin RNA II kit (MACHEREY-NAGEL GmbH & Co. KG, Düren, Germany), and an iScript cDNA Synthesis Kit and iTaq Universal SYBR Green Supermix were used for cDNA synthesis and qPCR analysis, respectively (Bio-Rad Laboratories Inc., Hercules, CA, USA). 3-(4,5-Dimethylthiazol-2-yl)-2,5-diphenyltetrazolium bromide (MTT) was purchased from Sigma-Aldrich (St. Louis, Mo., USA). Folin-Ciocalteu's phenol reagent, 1,1-diphenyl-2-picrylhydrazyl (DPPH), 6-hydroxyl-2,5,7,8-tetramethyl-chroman-2-carboxylic acid (Trolox), 2,2-Azobis(2-methylpropionamidine) dihydrochloride (AAPH), fluorescein, gallic acid, rutin, phenolic acids (4-hydroxybenzoic, caffeic, chlorogenic, ferulic, gallic, *p*-coumaric, synapic, syringic, transcinnamic, and rosmarinic acids), quercetin, quercetin-3-O-glucoside, quercetin-3-O-rhamnoside, quercetin-3-O-galactoside, kaempferol, kaempferol-3-O-rutinoside, hesperetin, hesperidin pure standards (>99.5% purity) in powder form, and HPLC-grade solvents were purchased from Sigma-Aldrich. All standards were prepared as stock solutions at 1 mg/mL in methanol and stored in the dark at -18°C for less than three months.

### 2.2. Preparation of Plant Extract

*Salvia sagittata* Ruiz & Pav (SS) plants were collected, according to previous authorization of the Ministry of the Environment (N. 003-IC-DPACH-MAE-2018-F), in Riobamba, Ecuador, on May 2016. The plants were identified and certified by Escuela Superior Politecnica de Chimborazo Herbarium, Riobamba, Ecuador, and a voucher specimen was deposited (N. 3342). Dried leaves (300 g) were ground and extracted with 96% ethanol for 48 h at room temperature. After filtration, the solvent was evaporated using a rotary vacuum evaporator (Büchi, Ch-9230, Flawil, Switzerland) and dried in a vacuum at 40°C to obtain the ethanolic extract with a yield of 6.18%. For experiments, the dry extract was dissolved in ethanol. The stock solution (20 mg/mL) was further used for HPLC analysis or diluted in culture media and membrane filtered by a 0.2 *μ*m Millipore filter (Millipore, Darmstadt, Germany).

### 2.3. Cell Culture and Treatments

Porcine aortic endothelial cells (pAECs) were isolated and maintained as previously described by Bernardini et al. [29]. Cells were seeded and routinely cultured in T25 tissue culture flasks (4 × 10^5^ cells/flask) (T 25-Falcon, Becton-Dickinson, Franklin Lakes, NJ, USA). Successive experiments were conducted in 96-well plates (cell viability and anti-inflammatory test) or 24-well plates (qPCR and Western blot) (both by Becton-Dickinson) with confluent cultures. Cells were cultured in hESFM and added with 5% FBS and 1x antimicrobial/antimycotic solution in a 5% CO_2_ atmosphere at 38.5°C.

The SSEE stock solution was diluted in the culture medium to obtain the desired concentrations (1–200 *μ*g/mL) for cell exposure. Ethanol (1%) was used as the vehicle.

### 2.4. Cell Viability

Viability was determined using the MTT assay. Briefly, pAECs (sixth passage) were seeded in 96-well culture plates at a density of 3 × 10^3^ cells/well and incubated for 24 h. Then, the media were replaced with hESFM containing 5% FBS and increasing SSEE doses (1, 10, 50, 100, and 200 *μ*g/mL) and incubated for another 24 h at 38.5°C. Next, the MTT solution (5 mg/mL in PBS) was added to a final concentration of 0.5 mg/mL and then incubated for 2 h at 38.5°C, followed by the addition of 0.1 mL MTT solubilisation solution. The formazan absorbance (Abs) was determined at 570 nm, using Infinite® F50/Robotic absorbance microplate readers from TECAN Life Sciences (Männedorf, Switzerland).

### 2.5. Cell Cycle

The medium of pAEC confluence (sixth passage) was replaced with hESFM containing 5% FBS and increasing SSEE doses (1, 10, 50, and 100 *μ*g/mL). After 24 h, pAECs were harvested and washed once in 5 mL of PBS, and 1 mL/10^6^ cells of 70% ice-cold ethanol was added drop by drop with continuous vortexing. The single cell suspension was fixed at 4°C for 24 h. Then, the cells were washed with 10 mL of PBS, and the cellular pellet was treated with 1 mL/10^6^ cells of staining solution containing PBS, 50 *μ*g/mL of PI, and 100 *μ*g/mL RNase A/T1 for 20 min in the dark at r.t. Cell distribution in cell cycle phases was analyzed by MACSQuant® Analyzer 10 and Flowlogic software (Miltenyi Biotec, Bergisch Gladbach, Germany). The Dean-Jett-Fox univariate model was used for this analysis.

### 2.6. In Vitro Tube Formation Assay

*In vitro* tube formation assay was performed as previously described [27]. Briefly, the experiments were carried out using an 8-slide-chamber glass (BD Falcon, Bedford, MA, USA) coated with an undiluted Geltrex™ LDEV-Free Reduced Growth Factor Basement Membrane Matrix. Firstly, the extracellular matrix coating was carried out 1 h before the seeding in a humidified incubator, at 38.5°C and 5% CO_2_. Then pAECs (seventh passage) (8 × 10^4^cells/well) were seeded with increasing SSEE doses (1, 10, 25, 50, and 100 *μ*g/mL) for 18 h.

At the end of the experimental time, images were acquired using a digital camera installed on a Nikon contrast phase microscope (Nikon, Yokohama, Japan) and analyzed by open software ImageJ 64.

### 2.7. Cell Viability after LPS Treatment

Briefly, pAECs (sixth passage) were seeded in 96-well culture plates at a density of 3 × 10^3^ cells/well and incubated for 24 h, then exposed to different SSEE concentrations (1, 10, and 100 *μ*g/mL) in the presence of LPS (25 *μ*g/mL) and incubated for another 24 h at 38.5°C.

The MTT solution was added to a final concentration of 0.5 mg/mL and then incubated for 2 h at 38.5°C followed by the addition of 0.1 mL of dimethyl sulfoxide to dissolve the MTT-formazan. The amount of MTT-formazan was then determined by measuring Abs at 570 nm.

### 2.8. Quantitative Real-Time PCR for IL-6, IL-8, and HO-1

pAECs (seventh passage) were seeded in a 24-well plate (approximately 4 × 10^4^ cells/well), incubated until confluence, and then exposed to different concentrations of SSEE (1, 10, and 100 *μ*g/mL) in the absence or presence of LPS (25 *μ*g/mL). RNA extraction was performed using the TRIzol reagent and NucleoSpin RNA II kit. After 24 h, the cells were harvested and lysed using 1 mL TRIzol reagent and mixed by vortex (3 min), and then, 200 *μ*L of chloroform was added to the suspension and mixed well. After incubation at room temperature (10 min), the samples were centrifuged (12000 g for 10 min) and the aqueous phase was recovered. An equal volume of absolute ethanol (99%) was added, and the resulting solution was applied to the NucleoSpin RNA Column. RNA was then purified according to the manufacturer's instructions. After spectrophotometric quantification, total RNA (250 ng) was reverse-transcripted to cDNA using the iScript cDNA Synthesis Kit in a final volume of 20 *μ*L.

Swine primers were designed using Beacon Designer 2.07 (PREMIER Biosoft International, Palo Alto, CA, USA). Primer sequences, expected PCR product lengths, and accession numbers in the NCBI database are shown in Table 1.

To evaluate gene expression profiles, the quantitative real-time PCR (qPCR) was performed in a CFX96 thermal cycler (Bio-Rad) using a multiplex real-time reaction for reference genes (glyceraldehyde-3-phosphate dehydrogenase, GAPDH; hypoxanthine phosphoribosyltransferase, HPRT; *β*-Actin, *β*-ACT) and using TaqMan probes and SYBR green detection for the target genes (interleukin-6, IL-6; interleukin-8, IL-8; Heme Oxygenase-1, HO-1). All amplification reactions were performed in 20 *μ*L and analyzed in duplicates (10 *μ*L/well). The multiplex PCR contained the following: 10 *μ*L of iTaqMan Probes Supermix (Bio-Rad), 1 *μ*L of forward and reverse primers (5 *μ*M each) of each reference gene, 0.8 *μ*L of iTaqMan probes (5 *μ*M) of each reference gene, 2 *μ*L of cDNA, and 2.6 *μ*L of water. The following temperature profiling was used: initial denaturation at 95°C for 30 seconds followed by 40 cycles of 95°C for 5 seconds and 60°C for 30 seconds.

The SYBR Green reaction contained the following: 10 *μ*L of iQ SYBR Green Supermix (Bio-Rad), 0.8 *μ*L of forward and reverse primers (5 *μ*M each) of each target gene, 2 *μ*L of cDNA, and 7.2 *μ*L of water. The real-time program included an initial denaturation period of 1.5 min at 95°C, 40 cycles at 95°C for 15 s, and 60°C for 30 s, followed by a melting step with ramping from 55°C to 95°C at a rate of 0.5°C/10 s.

The specificity of the amplified PCR products was confirmed by agarose gel electrophoresis and melting curve analysis.

The relative expressions of the studied genes were normalized based on the geometric mean of the three reference genes. The relative mRNA expression of the tested genes was evaluated as a fold of increase using the 2^-ΔΔCT^ method [30] and referred to pAECs cultured under the standard condition (control).

### 2.9. Western Blot for HO-1

Western blot for HO-1 was performed as previously described [24]. Briefly, pAECs (seventh passage), treated in the same manner as mentioned above, were washed twice with ice-cold PBS, harvested, and lysed in SDS solution (Tris-HCl 50 mM; pH 6.8; SDS 2%; glycerol 5%). After quantitative determination of the protein content by a Protein Assay Kit (TP0300, Sigma-Aldrich), aliquots containing 20 *μ*g of proteins were separated on NuPAGE 4-12% Bis-Tris gel for 45 min at 200 V and electrotransferred onto a nitrocellulose membrane. Blots were washed in PBS, and protein transfer was checked by staining the nitrocellulose membranes with 0.2% Ponceau S. After blocking the nonspecific binding with 5% nonfat milk in PBS-T20 (PBS-0.1% Tween-20) at room temperature for 1 h, membranes were incubated with a 1 : 1000 dilution of anti-HO-1 rabbit polyclonal antibody (SPA-896, StressGen Biotechnologies Corp., Victoria, BC, Canada) overnight at 4°C.

After several washings with PBS-T20, membranes were incubated with the secondary biotin-conjugate antibody and then with a 1 : 1000 dilution of an antibiotin horseradish peroxidase- (HRP-) linked antibody. The Western blots were developed using a chemiluminescent substrate (Clarity Western Substrate, Bio-Rad) according to the manufacturer's instructions. The intensity of the luminescent signal of the resultant bands was determined by the ChemiDoc Instrument using Lab Image Software (Bio-Rad).

In order to normalize the HO-1 data on the housekeeping protein, the membranes were stripped and reprobed for housekeeping *β*-tubulin (1 : 500 sc-5274 Santa Cruz Biotechnology Inc., Santa Cruz, CA, USA). The relative protein content (HO-1/*β*-tubulin) was expressed as arbitrary units (AUs).

### 2.10. Antioxidant Activity Assays

Antioxidant activity (AA) of SSEE was measured by the ORAC and DPPH assays. The ORAC assay was performed in an automated plate reader (Victor 3, PerkinElmer, Turku, Finland) with 96-well plates, according to Ou et al. [34] with some modifications. All reagents were freshly prepared before the assay. In each well, 210 *μ*L of fluorescein (10 nM) and 35 *μ*L of a sample, blank (10 mM phosphate buffer, pH 7.4), or standard (Trolox in the range 1-50 *μ*M) were placed. The plate was heated to 37°C for 10 min, and then, 35 *μ*L of AAPH (240 mM) was added, immediately before beginning fluorescence (FL) measurement. Relative FL intensity was monitored at 1.5 min intervals until it was less than 5% of the initial reading value. Final ORAC values were calculated by using a regression equation between the Trolox concentration and the net area under the FL decay curve and were expressed as mmol Trolox equivalents per g of extract or per g of plant material (DW).

The DPPH assay was done according to the method of Brand-Williams et al. [35] with some modifications. A stock solution was prepared by dissolving 24 mg DPPH with 100 mL methanol and then storing at -20°C until needed. The working solution was prepared by mixing 10 mL stock solution with 45 mL methanol to obtain an Abs of 1.1 ± 0.02 units at 515 nm. 150 *μ*L of SSEE was allowed to react with 2850 *μ*L of the DPPH solution for 24 h in the dark. Abs measurements were carried out at 515 nm. Results were determined from the regression equation of the Trolox calibration curve in the range of 25-500 *μ*M and expressed as mmol TE per g of extract or per g of plant material (DW).

### 2.11. Total Phenol Content and Total Flavonoid Content

Total Phenol Content (TPC) was determined using the Folin-Ciocalteu method [36]. 50 *μ*L of diluted extract was mixed with 250 *μ*L of a tenfold-diluted Folin-Ciocalteu phenol reagent. After 1 min, 800 *μ*L of 30% sodium carbonate solution was added to the mixture, shaken thoroughly, and diluted to 1.6 mL by adding 500 *μ*L of distilled water. The mixture was allowed to stand for 40 min at r.t., and the blue colour formed was measured at 700 nm using a UV-VIS spectrophotometer (V-630 Jasco, Jasco Europe S.r.l., Cremella, Italy). A calibration curve of gallic acid (ranging from 5 to 500 *μ*g/mL) was prepared, and the results, determined from the regression equation of the calibration curve, were expressed as mg of Gallic Acid Equivalents (GAE) per g of extract or per g of plant material (DW).

The total flavonoid content (TFC) was determined according to Zhishen et al. [37] with some modifications. 500 *μ*L of extract was diluted to 5 mL with distilled water, 300 *μ*L of 5% NaNO_2_ was added, and the mixture was mixed well. After 5 min, 3 mL of a 10% AlCl_3_ solution was added. After 6 min, 2 mL of a 1 M NaOH solution was added, and the total volume was made up to 10 mL with distilled water. Absorbance was measured against a blank at 510 nm. Rutin was used as the standard for the calibration curve. TFC was calculated using the regression equation based on the calibration curve, and the results were expressed as mmol of Rutin Equivalents (RE) per g of extract or per g of plant material (DW).

### 2.12. HPLC-DAD Determination of Phenolic Acids and Flavonoids

HPLC-DAD determination of phenolic acids and flavonoids was performed as previously described [38]. 20 *μ*L of SSEE was injected into the HPLC system (Jasco Italy; PU-4180 pump, MD-4015 PDA detector, AS-4050 autosampler). The stationary phase was an Agilent (Santa Clara, CA, USA) ZORBAX Eclipse Plus C18 reversed-phase column (100 mm × 3 mm I.D., 3.5 *μ*m). The chromatographic method for the analysis of phenolic acids was adapted from Mattila and Kumpulainen [39]. Gradient elution was carried out with a mixture of acidic phosphate buffer (50 mM, pH 2.5) and acetonitrile flowing at 0.7 mL/min. Signals at 254, 280, and 329 nm were used for analyte quantitation. The recovery values of phenolic acids in spiked samples ranged from 78.8 to 92.2% (RSD < 9.8%, *n* = 6). The chromatographic method for the analysis of flavonoids was adapted from Wojdyło et al. [40]. Gradient elution was carried out with a mixture of 4.5% formic acid and acetonitrile. Runs were monitored at 280 nm for flavan-3-ols and 360 nm for flavonol glycosides. Retention times and spectra were compared with those of pure standards. Calibration curves were constructed for all standards at concentrations ranging from 1.0 to 100.0 ppm (*r*^2^ ≤ 0.9998). Results were expressed as mg/g extract or per g of plant material (DW).

## 3. Statistical Analysis

Each treatment was replicated three or eight times (viability and anti-inflammatory tests) in three independent experiments. Data were analysed by a one-way analysis of variance (ANOVA) followed by the post hoc Tuckey comparison Test. Differences of at least *p* < 0.05 were considered significant. Statistical analysis was carried out using GraphPad Prism 7 software.

## 4. Results and Discussion

### 4.1. Effect of SSEE on pAEC Viability and Angiogenesis

A large number of plants of the Ecuadorian flora are used for medicinal purposes. Nevertheless, scientific evidence supporting their use is still scarce. For this reason, a study was planned on the antiangiogenic and antioxidant activity and on phytochemical composition of *Salvia sagittata*, an endemic plant used in Ecuadorian Traditional Medicine. This choice was corroborated by the fact that several *Salvia* species were demonstrated to possess a protective effect against different external agents [40, 41].

**Figure 1:**
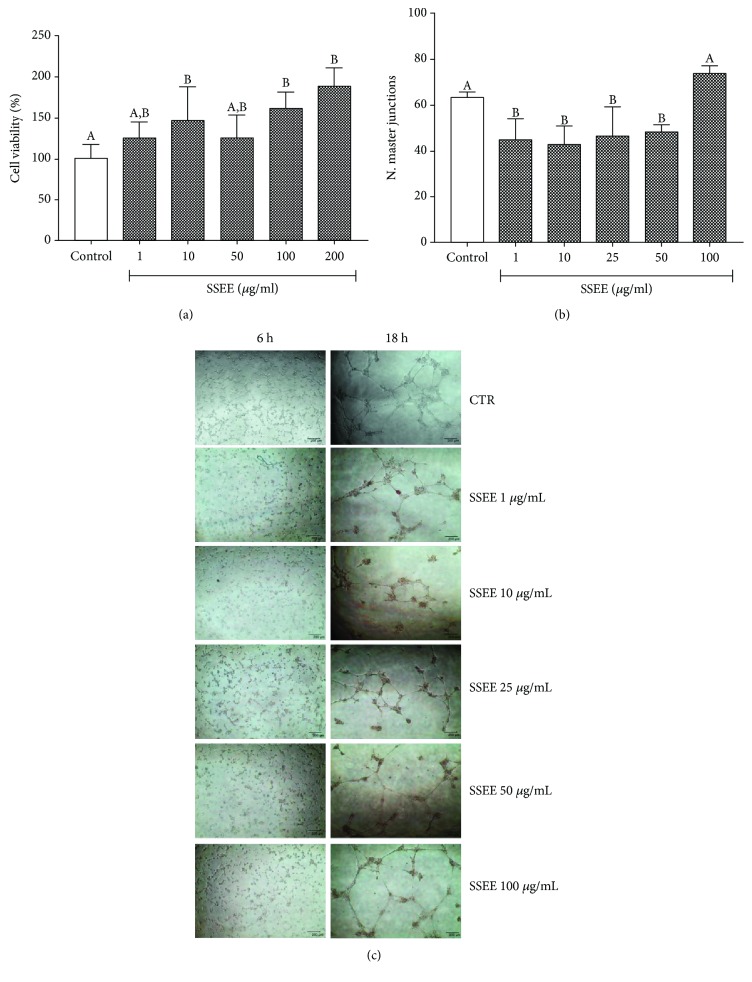
Effects of *S*SEE on pAEC physiology. Cells were treated with different concentrations of SSEE for (a) 24 h for cell viability and (b) 18 h for angiogenesis. Cell network formations were recorded at 6 h and 18 h after treatment (c). Data shown are representative of 8 (a) or 3 (b) replicates in at least three independent experiments. Each bar represents mean ± S.D. Different letters above the bars indicate significant differences (*p* < 0.05, ANOVA, post hoc Tukey's test).

Based on the traditional uses of the plant, it was decided to focus the biological tests on anti-inflammatory activity. A preliminary screening aimed at verifying the safety of SSEE was carried out. Treatment of pAECs with SSEE for 24 h did not negatively affect cell viability at any concentration tested (Figure 1(a)). Cells possessed a standard cell cycle for diploid cells (data not shown). Thus, SSEE does not seem to induce any cytotoxicity effect on pAECs in the concentration range examined. These results are in agreement with other researches in which different *Salvia* species did not affect cell viability [42]. After this preliminary assay, pAECs' angiogenesis was examined by an *in vitro* extracellular matrix-based assay. As a result, the capacity of cells to assemble a tube network formation was reduced after treatment with SSEE at 1, 10, 25, and 50 *μ*g/mL (Figure 1(b) and Figure 1(c)).

### 4.2. Effect of SSEE on LPS-Induced Cell Death and Cytokine Expression

**Figure 2:**
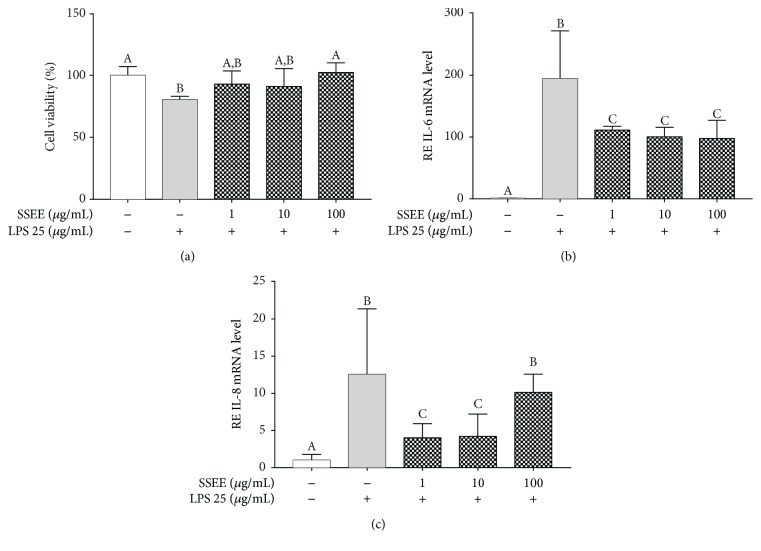
Effects of SSEE on LPS-induced pAEC damage. (a) Effect of SSEE on LPS-induced cytotoxicity. Each bar represents mean ± S.D. Effect of SSEE on (b) IL-6 and (c) IL-8 mRNA expression. Relative expression (RE) was calculated as the fold of change with respect to the control cells, and the error bars represent the range of relative gene expression. Data shown are representative of at least three independent experiments. Different letters above the bars indicate significant differences (*p* < 0.05, ANOVA, post hoc Tukey's test).

Inflammation and endothelial cells are closely related. In fact, in an inflammatory process, endothelial cells trigger the transcription of genes such as TNFR or TLR4 that activates the NF-*κ*B pathway and induces the expression of adhesive receptors (VCAM-1, E-selectin, and ICAM-1), procoagulant proteins (TF, PAI-1), cytokines, chemokines, and protective proteins [17]. Therefore, the anti-inflammatory activity of SSEE on pAECs was evaluated by an endothelial LPS inflammatory model [27, 28]. Firstly, it was decided to assess a possible SSEE protective effect against LPS damage through a MTT assay. LPS exerted an evident cytotoxic effect, producing a significant 20% reduction of pAEC viability (Figure 2(a)) SSEE significantly reduced LPS-induced cytotoxicity, restoring the basal levels at 100 *μ*g/mL concentration (Figure 2(a)).

Given the well-documented relationship between the inflammation process and oxidative stress [43], the *in vitro* antioxidant activity (AA) of the extract was evaluated by two assays, based on two different mechanisms: the ORAC assay, which is based on the hydrogen-atom transfer (HAT) mechanism, and the DPPH assay, which is an electron transfer assay. As it can be seen from Table 2, the AA values of the extract were rather similar in the two assays, being 0.10 and 0.11 mmol TE/g DW, respectively. Even though it is extremely difficult to compare and to interpret data on the AA of plant extracts, due to the wide number of factors affecting the activity (extract preparation procedure, test method used, etc.), the values of the extract resulting from both the DPPH and ORAC assays were very close to those reported for other crude plant extracts prepared in a similar way [44].

The antioxidant activity and protective effect of SSEE against LPS might confirm its anti-inflammatory activity and justify the traditional *Salvia sagittata* uses. Nevertheless, to gain insight into the molecular mechanisms involved in mediating these responses, it was decided to investigate how SSEE could influence some of the main inflammatory markers and protective molecules expressed by endothelial cells at both the transcription and proteomic levels.

IL-6 and IL-8 are stress-responsive proinflammatory chemokines that play a pivotal role in the pathogenesis of different acute inflammatory conditions [44, 45]. They can be synthesized by different cell types; IL-6 activates the differentiation of cytotoxic T cells and the monocyte and induces angiogenesis and increases vascular permeability [46, 47], while IL-8 is a potent leukocyte and fibroblast chemoattractant/activator and it is closely associated with endothelial permeability, inflammatory recruitment, and release of proinflammatory mediators [48].

As can be seen in Figures 2(b) and 2(c), LPS induced a significant increase of both the IL-6 and the IL-8 mRNA expression after 24 h of treatment. SSEE at all tested concentrations was able to revert the LPS-induced IL-6 gene expression increase (Figure 2(b)) and to revert that of IL-8 in the 1-10 *μ*g/mL range (Figure 2(c)).

This is in agreement with other *in vivo* and *in vitro* reports on other species of the *Salvia* genus. Yue et al. [49] have reported that *S. miltiorrhiza* induced a reduction of cytokine expression, thus alleviating liver inflammation. In a similar way, Gao et al. [50] have reported the reduction of nitric oxide, tumor necrosis factor (TNF-*α*), and IL-6 secretion in RAW264.7 macrophages by a novel compound isolated from S. *miltiorrhiza* in a LPS inflammatory model.

### 4.3. Effect of SSEE on HO-1 Expression

**Figure 3:**
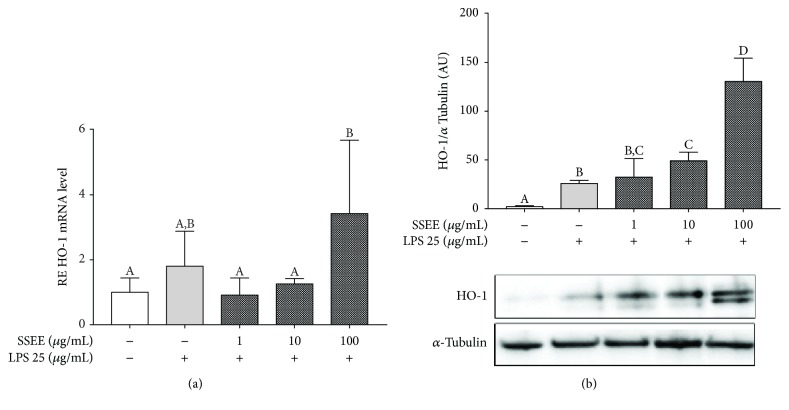
Effects of *SSEE* on the HO-1 expression in LPS-induced pAEC damage. (a) Expression of the HO-1 mRNA relative expression was calculated as the fold of change with respect to the control cells, and the error bar represents the range of relative expression. (b) Representative Western blot of HO-1 and relative housekeeping *α*-tubulin. Data shown are representative of three replicates in at least three independent experiments. Each bar represents mean ± S.D. Different letters above the bars indicate significant differences (*p* < 0.05, ANOVA, post hoc Tukey's test). AU: arbitrary unit.

In an inflammatory process, to avoid endothelial dysfunction, there is a tight balance between inflammatory and protective molecules. Recent findings indicated that HO-1, initially studied for its ability to degrade heme, is a key regulator molecule of endothelial cell function providing an important cellular defense mechanism against tissue injury [50, 51]. Moreover, during chronic inflammation, HO-1 performs a double function inhibiting leukocyte infiltration and promoting VEGF-driven noninflammatory angiogenesis that facilitates tissue repair [52]. For this reason, the HO-1 gene expression and protein level were evaluated by RT-PCR and Western blot, respectively. Compared to the control, the SSEE treatment at the highest tested level increased the HO-1 gene expression (Figure 3(a)); concerning the protein level, a clearer dose-dependent response was observed (Figure 3(b)). Although LPS itself did induce an increase in HO-1 levels [29], upon SSEE treatment, the protein level was even higher, and this suggests a possible correlation with its anti-inflammatory properties towards interleukins.

### 4.4. Phytochemical Investigation of SSEE

Phytochemical investigation of the polyphenolic composition was done through both spectrophotometric assays and HPLC-DAD (Table 2). TPC and TFC suggest that SSEE was rich in polyphenols, and flavonoids represented more than 65% of polyphenol structures. TPC through the Folin-Ciocalteu procedure was investigated in a wide array of medicinal plants, and values ranging from 9 to 183 mg GAE/g DW in plants belonging to different botanical families were reported. Considering the slight differences in the extract preparation and the different species investigated by these authors, the TPC content of the *Salvia sagittata* extract (10.19 mg GAE/g DW) turned out to be very close to that reported by Kähkönen et al. [53] for *Thymus vulgaris* methanolic extract (9 mg GAE/g DW).

**Figure 4:**
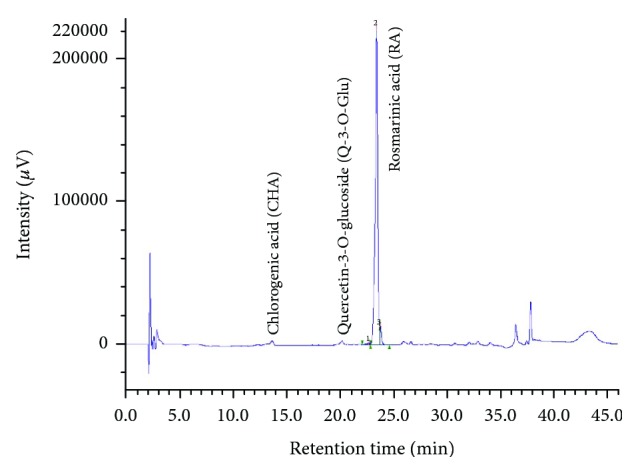
The HPLC profile of SSEE showing the main phenolic compounds identified.

HPLC-DAD analysis showed that the major phenolic acid-derivative compound in the extract was rosmarinic acid (RA) (Figure 4), reaching about 50% of TPC. Other phenolic compounds were, in decreasing order, chlorogenic acid, quercetin-3-O-glucoside, hesperetin, cinnamic acid, and syringic acid, the latter being present only in trace amounts (Table 2). These results are in agreement with a recent phytochemical screening carried out on three plant extracts belonging to the *Lamiaceae* family by Cocan et al. [54], which demonstrates that RA represents the major phenolic acid compound of an ethanolic extract from *Salvia officinalis* (L.) leaves.

RA has been previously demonstrated to exert anti-inflammatory and antiangiogenic activity, and its mechanism of action has been deeply investigated both *in vitro* and *in vivo*. Huang and Zheng [55] reported that this phenolic compound reduced the H_2_O_2_-dependent VEGF expression and IL-8 release in human umbilical vein endothelial cells, as well as intracellular ROS levels. In human leukemia cells, RA treatment significantly sensitizes TNF-*α*-induced apoptosis through the suppression of NF-*κ*B and ROS generation [56], and in a tumor-bearing mice model, a tumor growth-suppressing activity was demonstrated [57]. Thus, most biological activities of RA, including the neuroprotective [58] and hepatoprotective [59] ones, have been related to its marked antioxidant properties, deriving from its ability to act as a lipid peroxidation inhibitor and ROS scavenger [60].

Thus, it is possible to hypothesize that this cinnamic acid derivative gives the main contribution to the anti-inflammatory effects of the ethanolic extract of *Salvia sagittata* observed in this study, although the role of other phenolic compounds, not identified in our analysis, cannot be excluded. Moreover, a synergistic action among all chemical components can also explain the biological activity of the extract.

In conclusion, the results obtained here represent the first evidence on phytochemical aspects and anti-inflammatory activity of the *Salvia sagittata* extract, which can justify the use of this plant in the Ecuadorian Traditional Medicine.

## Figures and Tables

**Table 1:** Primer sequences used for quantitative real-time polymerase chain reaction analysis.

Gene	Sequence (5′-3′)	PCR product (bp)	GenBank accession number	Reference
HO-1	For: CGCTCCCGAATGAACAC Rev: GCTCCTGCACCTCCTC	112	NM_001004027	Bernardini et al. [31]

IL-8	For: AGGACCAGAGCCAGGAAGAGAC Rev: TGGAAAGGTGTGGAATGCGTATTTATG	203	AB057440.1	Present study

IL-6	For: AGCAAGGAGGTACTGGCAGAAAACAAC Rev: GTGGTGATTCTCATCAAGCAGGTCTCC	110	AF518322.1	Zannoni et al. [32]

GAPDH	For: ACATGGCCTCCAAGGAGTAAGA Rev: GATCGAGTTGGGGCTGTGACT Probe: HEX-CCACCAACCCCAGCAAGAGCACGC-BHQ1	106	NM_001206359	Duvigneau et al. [33]

HPRT	For: ATCATTATGCCGAGGATTTGGAAA Rev: TGGCCTCCCATCTCTTTCATC Probe: Tx-Red-CGAGCAAGCCGTTCAGTCCTGTCC-BQ2	102	NM_001032376	Present study

*β*-ACT	For: CTCGATCATGAAGTGCGACGT Rev: GTGATCTCCTTCTGCATCCTGTC Probe: FAM-ATCAGGAAGGACCTCTACGCCAACACGG-BHQ1	114	KU672525.1	Duvigneau et al. [33]

**Table 2:** Antioxidant activity (AA), TPC, TFC, phenolic acids, and flavonoid content (expressed as mg/g) in SSEE. Data are the mean ± S.E. of three technical determinations.

Assays or compounds	Concentration referred to	
	Ethanolic plant extract	Plant dry weight
AA		
ORAC (mmol TE/g)	1.85 ± 0.15	0.11 ± 0.09
DPPH (mmol TE/g)	1.57 ± 0.11	0.097 ± 0.007
TPC	164.95 ± 8.57	10.19 ± 0.53
TFC	109.13 ± 5.23	6.74 ± 0.32
RA	84.76 ± 9.32	5.23 ± 0.57
HESP	0.2 ± 0.02	0.012 ± 0.001
Q-3-O-GLU	0.7 ± 0.04	0.043 ± 0.004
CHA	1.32 ± 0.16	0.08 ± 0.09
CA	0.17 ± 0.02	0.010 ± 0.001
SA	0.02 ± 0.003	0.001 ± 0.0001

RA: rosmarinic acid; HESP: hesperetin; Q-3-*O*-GLU: quercetin-3-O-glucoside; CHA: chlorogenic acid; CA: caffeic acid; SA: syringic acid.

## Data Availability

The original data used to support the findings of this study are available from the corresponding author upon request.
